# Polygenic risk scores for prediction of atrial fibrillation

**DOI:** 10.1007/s12471-022-01755-y

**Published:** 2022-12-20

**Authors:** Maryam Kavousi, Patrick T. Ellinor

**Affiliations:** 1 Department of Epidemiology, Erasmus MC, University Medical Center Rotterdam, Rotterdam, The Netherlands; 2 Cardiovascular Research Center, Massachusetts General Hospital, Boston, MA USA; 3 Cardiovascular Disease Initiative, Broad Institute of MIT and Harvard, Cambridge, MA USA

As the most common sustained cardiac arrhythmia, atrial fibrillation (AF) is an important contributor to population morbidity and mortality. AF is a complex disorder resulting from the interplay of a multitude of genetic, epigenetic and environmental factors. Epidemiological studies have documented a substantial genetic component for AF; with heritability estimates as large as 22% based on common genetic variants in individuals of European ancestry [1].

Common diseases typically have a polygenic inheritance that involves numerous common genetic variants with a small effect, the cumulative effect of which can play a greater role than rare monogenic mutations [2]. As a single variant is not informative enough for assessing disease risk, polygenic risk scores (PRS) provide a measure of genetic susceptibility conferred by the combination of many disease-associated risk variants. A PRS for AF can be used to identify individuals at higher risk for AF and its associated complications. An initial study by the AFGen Consortium examined seven different AF-PRSs containing between 11–719 single-nucleotide variants (SNVs). Individuals in the highest AF-PRS quartile had a 1.28-fold (719 SNVs) to 1.67-fold (25 SNVs) larger hazard for AF [3]. While the AF-PRSs were associated with incident AF beyond clinical AF risk factors as well as with cardio-embolic stroke in age- and sex-adjusted analyses, improvements in discrimination were small [3]. In a recent study in the UK Biobank, a PRS using over 6.7 million SNVs, identified 6.1% of the population that were at over 3‑fold risk for prevalent and incident AF. The risk was deemed similar to that for monogenic diseases [2].

Despite the potential promise of AF-PRS, challenges remain in how to interpret and integrate PRS results into clinical decision making. Ultimately, it will be important to place the PRS results for an individual into a broader context which involves each person’s characteristics, clinical profile, co-morbidities, and preferences. In one recent application of an AF-PRS in the Framingham Heart Study, individuals in the lowest tertile of clinical and polygenic risk had a lifetime AF risk of 22%. In contrast, individuals in the lowest tertile of clinical risk but highest tertile of polygenic risk had a 44% lifetime risk for AF. Of note, 11% of individuals in this study, while deemed to be at high clinical risk, were at the lowest tertile of polygenic risk [4]. The observed interplay between the AF-PRS and clinical AF risk in this study also poses new questions as to: 1) what the clinically actionable risk or high-risk thresholds for AF prevention are, and 2) how to devise prevention strategies among those with a low AF-PRS but deemed to be at high AF risk based on clinical prediction algorithms.

Differences in genetic variant frequencies and linkage disequilibrium patterns between various populations as well as differences in heritability for the same phenotype across populations have been reported. For example, genetic heterogeneity in AF susceptibility loci across racial/ethnic groups exists [1]. These findings, both for AF and other common diseases, will require large-scale genome-wide association studies (GWAS) in diverse human populations and warrant consideration of genomic differences between populations when applying risk estimation models. Similarly, sex differences in AF pathophysiology and prognosis have been suggested [5]. However, genetic contributions to sex differences in AF risk have not been widely investigated. Future studies should consider sex differences in genetic predisposition to AF and a PRS should not be assumed to perform equally well in both sexes for AF prediction.

Lastly, efforts are warranted to determine whether AF-PRS may improve identification of subclinical AF, discrimination between various AF patterns or AF progression, or distinguishing between various underlying mechanisms for subsequent stroke or heart failure. For a polygenic complex condition as AF, the aetiologically informed risk classification can lead to improvements in prevention, diagnosis, treatment and prognosis.
